# Challenges in optimizing the histidine rich protein 2-detecting rapid diagnostic test

**DOI:** 10.1186/1475-2875-13-S1-P20

**Published:** 2014-09-22

**Authors:** Robert Burton, Kathy Tietje, Kelly Ebels, Paul LaBarre

**Affiliations:** 1 PATH, Seattle, WA, USA

## 

Malaria programs aimed at eliminating *Plasmodium falciparum* (Pf) incorporate active infection detection strategies to target the subclinical transmission reservoir. Currently available immunochromatographic lateral flow tests lack the sensitivity required for Pf malaria elimination; the limit of detection (LOD) of these rapid diagnostic tests (RDTs) is above that which is required to identify all transmissible infections. Malaria RDTs with a significantly improved LOD would enable more effective elimination interventions while retaining the critical advantages of low cost, ease of use, and rural deployment capability. Histidinerich protein 2 (HRP2) is a high-priority target analyte for identifying individuals at risk of transmitting Pf. HRP2 protein is secreted by Pf parasites in relatively high concentrations, is widely conserved among Pf strains, and persists in human plasma for up to four weeks. These characteristics allow HRP2 antigen detection even in the absence of circulating parasites during cyclical sequestration. However, the highly polymorphic nature of HRP2 makes optimization of RDTs challenging. Current HRP2-detecting RDT reagents primarily target Type 2 (AHHAHHAAD) and Type 7 (AHHAAD) HRP2 motifs, and independent antibody discovery confirms the most common selection of a C1-13/PTL-3 antibody conjugate pair yields the best RDT performance for the majority of parasite strains. In scenarios where malaria transmission rapidly decreases, exclusive use of a diagnostic whose sensitivity varies with the number of HRP2 epitope repeats can result in single axis evolutionary pressure, increasing selection for less-detectable parasite strains. To evaluate the feasibility of developing an HRP2-based RDT that is highly effective in mass testing and treatment programs, we have assessed the technical characteristics of HRP2 reagents. Our aim is to facilitate the development of improved malaria HRP2 RDTs by: (1) incorporating an understanding of HRP2 structure-function relationships and their impact on epitope-antibody interactions and adaptation, (2) correlating HRP2 concentration levels (in pg/mL) with traditional malaria diagnostic metrics (parasites/mL) for comparative evaluation of tests, and (3) predicting optimal capture reagents to improve and sustain HRP2 RDT performance. Based on our ongoing analysis, we present a product development strategy that combines capture reagent and platform innovations that can be used to make highly sensitive Pf malaria RDTs. In addition, we present criteria for the rational selection of HRP2 protein and parasite culture controls to support the product lifecycle from development through commercialization and scale-up.

